# Analyzing caesarean sections through the Robson classification in Nigeria: a prospective nationwide study in referral level facilities

**DOI:** 10.1016/j.eclinm.2025.103427

**Published:** 2025-09-01

**Authors:** Tina Lavin, Amaka N. Ocheke, Ana Pilar Betran, Abiodun S. Adeniran, Eziamaka Ezenkwele, Duum C. Nwachukwu, Luz Gibbons, Aisha Abdurrahman, Sulaiman Muhammad Deneji, Akpanika Chinyere, Adewale Ashimi, Rais Ibraheem, Odward Elusoji Jagun, Innocent Anayochukwu Ugwu, Olufemi Aworinde, Agada Egwu, Sunday Ochigbo, Timothy A.O. Oluwasola, Aniekan Abasiattai, Anthonia Inibokun Njoku, Lawal Magaji, Peter Aboyeji, Hadiza Galadanci, Calvin Chama, Saturday Etuk, Joseph Ikechebelu, Olubukola Adesina, Jamilu Tukur, Maternal and Perinatal Database for Quality, Equity and Dignity Network

**Affiliations:** a UNDP/UNFPA/UNICEF/WHO/World Bank Special Programme of Research, Development and Research Training in Human Reproduction Programme (HRP), Department of Sexual and Reproductive Health and Research, World Health Organization, Geneva, Switzerland; b Jos University Teaching Hospital, Jos, Nigeria; c Department of Obstetrics and Gynaecology, University of Ilorin Teaching Hospital, Ilorin, Nigeria; d University of Nigeria Teaching Hospital Enugu, Engu, Nigeria; e Federal Medical Centre Bida, Bida, Nigeria; f Institute for Clinical Effectiveness and Health Policy (IECS), Department of Mother & Child Health Research, Buenos Aires, Argentina; g Federal Teaching Hospital Katsina, Katsina, Nigeria; h Standard Specialist Hospital, Kano, Nigeria; i University of Calabar Teaching Hospital, Calabar, Nigeria; j Federal Medical Centre, Birnin, Kudu, Nigeria; k National Hospital Abuja, Federal Capital Territory, Nigeria; l Olabisi Onabanjo University Teaching Hospital, Nigeria; m Enugu State University Teaching Hospital, Enugu, Nigeria; n Bowen University Teaching Hospital, Oyo, Nigeria; o Federal Medical Centre Makurdi, Makurdi, Nigeria; p University College Hospital, Ibadan, Nigeria; q University of Uyo Teaching Hospital, Uyo, Nigeria; r Federal Medical Centre Irrua, Irrua, Nigeria; s African Center of Excellence for Population Health and Policy, Bayero University Kano, Nigeria; t Department of Obstetrics and Gynaecology, Abubakar Tafawa Balewa University Teaching Hospital, Bauchi, Nigeria; u Department of Obstetrics and Gynaecology, Nnamdi Azikiwe University Teaching Hospital, Nnewi, Nigeria; v Department of Obstetrics and Gynaecology, Aminu Kano Teaching Hospital, Kano, Nigeria

**Keywords:** Robson classification, Induction of labour, Caesarean section, Perinatal outcomes

## Abstract

**Background:**

Over the past 30 years, there has been increased concern on rising caesarean section rates. However, the absence of reliable data on appropriateness of caesarean section for women in many countries, including Nigeria, poses a significant obstacle to understanding the use of caesarean and the quality of care surrounding caesarean section. The objective of this study was to analyse the caesarean section rates in specific obstetric populations to better understand the appropriateness of caesarean section by Robson Group in 56 referral-level facilities across Nigeria.

**Methods:**

Data from 179,463 women who gave birth in 56 referral-level facilities across Nigeria between 1 September 2019 and 31 August 2022 were analysed using the Robson classification and interpreted using the WHO Implementation Manual.

**Findings:**

Of the 158,246 women classified by Robson, 52,221 (33%) had caesarean section. Women with previous caesarean section (Group 5) were the largest contributors to overall caesarean section rate, accounting for 27.1% of all caesarean sections. This was followed by women with preterm birth (Group 10–17.1%). Women with term induced labour or those who had a pre-labour caesarean section also made substantial contributions to overall caesarean section rate (Group 2 (nullipara women)–12.8%; Group 4 (multipara women)–12.0%). When examining caesarean section rate within specific obstetric populations, Group 2 and Group 4 (nullipara and multipara women without previous caesarean section) had particularly high caesarean section rates—84.0% and 77.7%, respectively. Most of these were pre-labour caesarean sections: 83.9% (5620/6702) in Group 2 and 90.6% (5676/6263) in Group 4, few women in these obstetric populations had labour induction (16.1% in Group 2; 9.4% in Group 4). Among nulliparous women undergoing pre-labour caesarean section the main indications were hypertensive disorders (18.9%) and suspected contracted/inadequate pelvis (13.2%). For multiparous women, hypertensive disorders (15.0%) and placental conditions (11.9%) were the leading indications. Group 2a and Group 4a (women who had induction of labour) also had high caesarean section rates–45.9% and 24.6%, respectively.

**Interpretation:**

This nationwide programme shows a high caesarean section rate among women with a previous caesarean section, highlighting the importance of appropriate caesarean section use in nulliparous women to prevent caesarean section in future pregnancies. Women with term pregnancies without previous caesarean section had a high rate of caesarean section while the rate of labour induction in the same population of women was low. Among women who had an induction of labour, a substantial proportion had a caesarean section. There may be an opportunity to reduce caesarean rate by strengthening strategies to identify women who are good candidates for induction of labour and by fostering an environment that supports safe and successful induction. A multi-faceted approach is needed including adequate training for health workers, creating a calm birthing environment, ensuring health workforce capacity to monitor women during labour induction and ensuring access to quality medications all within a the context of a well-functioning and well-financed health-care system.

**Funding:**

This work was funded by MSD for Mothers; and UNDP/UNFPA/UNICEF/WHO/World Bank Special Programme of Research, Development and Research Training in Human Reproduction Programme (HRP), a co-sponsored programme executed by the 10.13039/100004423World Health Organization (WHO).

**Research in context**

**Evidence before this study**

In the context of rising caesarean section rates, a shortage of dependable epidemiological data impedes a comprehensive understanding on appropriateness of caesarean section use. Several studies suggest that many LMICs may be grappling a two-fold challenge: the under-utilization of necessary interventions such as caesarean section, often delivered too late, and the unnecessary use of interventions often delivered too soon. This complex scenario has been described as “Too little, too late, too much, too soon”. However, the absence of reliable data makes it unclear where many countries are positioned on this continuum and where efforts should be directed.

The Robson Ten Group Classification System is widely employed for caesarean section audit. However, a recent systematic review found that there was no study in Nigeria using the Robson classification to analyse caesarean section across multiple facilities and an assessment at a national level was lacking.

**Added value of this study**

Around one-third of women across the participating facilities in Nigeria had a caesarean section. For women without previous caesarean section (Group 2 and Group 4) pre-labour caesarean section was used more frequently than in the multi-country observational studies in LMICs included in the WHO Robson Interpretation Manual (Group 2–84% compared to 39.9%; Group 4 77.7% compared to 23.7%). Induction of labour was less common than in the existing observational studies in LMICs -WHO MCS 4.5–15.5% of women had an induction of labour, compared to 3% in the current study. When induction of labour was used a high proportion of women had a subsequent caesarean section.

**Implications of all the available evidence**

The Robson classification can be applied using simple information that is routinely collected, and can serve as a tool to assess and monitor cesarean section rates helping healthcare providers and policymakers identify areas for improvement and implement strategies to promote appropriate and evidence-based obstetric care. An opportunity to optimize the use of interventions may be to target improved identification of women for induction of labour (in place of pre-labour caesarean sections) along with creating a supportive environment for successful induction. This includes incorporating elements such as dedicated staff time and health worker training, continuous labour and fetal monitoring, companionship during labour, creating a calm birthing environment for women and ensuring access to quality pharmaceuticals. Harmonized evidence-based guidelines from professional bodies on induction of labour in Nigeria under-pinning critical elements for successful induction are urgently needed. There is an opportunity for cross-facility learning as we observed that facilities who performed fewer pre-labour caesarean sections (and more inductions of labour), had a higher frequency of successful induction of labour. Targeted training and cross-learning between facilities that commonly perform induction of labour with high success and good outcomes for women and their babies coupled with providing a supportive environment for induction may increase confidence and provider preference for induction of labour.

The high facility caesarean section rate was in contrast to the low population caesarean section rate across Nigeria (2.7%), suggesting that many women still lack access to this life-saving intervention.

## Introduction

Over the last three decades, there has been growing concern about the increasing rate of caesarean section.^1^ However a dearth of reliable epidemiological data on mode of delivery and outcomes for women and babies, particularly in low- and middle-income countries (LMIC) hampers a comprehensive understanding of caesarean use and the quality of care surrounding caesarean section. A recent meta-analysis found over 1% mortality among women who birth by caesarean in sub-Saharan Africa, 100 times higher than in the United Kingdom.^2^ One in 10 mothers in sub-Saharan Africa who birth by caesarean section experience a stillbirth or early neonatal death.^2^ This may indicate limited access to safe and timely caesarean section as a substantial barrier to improving the outcomes of mothers and neonates. Paradoxically, many LMICs may be facing a dual challenge: the under-utilization of necessary interventions such as caesarean section, often delivered too late, and the unnecessary use of interventions often delivered too soon. This situation has been described as “Too little, too late, too much, too soon”.^3^ However without reliable and appropriately analyzed data, it is unclear where many countries are positioned on this continuum and where the effort should be made.

Caesarean section is a globally recognized maternal health-care indicator for access to obstetric services,^4^^,^^5^ and reflects both “Too little, too late” and “too much, too soon” — with wide disparities between and within countries.^1^^,^^6^ In many lower-income countries, providing safe cesarean section to appropriately selected women remains a major challenge to improving maternal and newborn outcomes.^7^ A recent study in Nigerian tertiary facilities found that ten-percent of women with severe maternal outcomes experienced treatment delays of more than 4 h.^8^ Another observational study reported suboptimal quality care in referral-level facilities, with delays contributing to 998 maternal deaths in the one year study.^9^ Conversely, an audit in 22 referral hospitals in Burkina Faso concluded that around 25% of the caesarean sections were not medically necessary.^10^

Recently, in the context of growing use, there has been greater attention on the appropriateness of caesarean section and how to optimize its use. To help assess the use of caesarean section, the WHO recommended in 2015 the Robson classification (also known as the 10-group classification) as a global standard for assessing, monitoring and comparing caesarean section rates both within healthcare facilities and between them. The system classifies all women into one of ten mutually exclusive categories which, as a set, is totally comprehensive. The categories are based on five basic obstetric characteristics that are routinely collected in facilities: parity, number of fetuses, previous caesarean section, onset of labour, gestational age, and fetal presentation.

A recent systematic review found that there was no study in Nigeria using the Robson classification to understand intra-facility caesarean section rates.^11^ Despite the low overall population caesarean section rate in Nigeria, estimated around 3% (approximately 200,000 caesareans are conducted yearly), it is estimated that an additional 542,000 would be needed to reach the 10% nationally needed to improve perinatal outcomes.^1^^,^^12^ Improving the understanding of caesarean section use, identifying which groups of women are most in need, and determining where overuse may occur is crucial for shaping policies, developing clinical protocols, and ultimately enhancing the quality of care and outcomes in this country.

The Maternal, Perinatal, Database for Quality, Equity and Dignity programme (MPD-4-QED Programme) presents an opportunity to generate reliable epidemiological data on the use of caesarean section, in a high mortality country, and to systematically analyse Robson categories. The MPD-4-QED Programme collected data from more than 195,000 women giving birth in a network of 54 referral level facilities providing in-patient services for obstetric and gynaecology in Nigeria between 1 September 2019 and 31 August 2022. The objective of this study was to assess the appropriateness of caesarean section in the participating referral facilities The specific objectives were: (1) To classify women using the Robson Classification and report the relative size of each Robson group, the caesarean section rate in each Robson group, and the absolute and relative contributions made by each to the overall caesarean section rate; and (2) To assess indications for caesarean section by Robson group.

## Methods

In partnership with the Nigeria Federal Ministry of Health, the World Health Organization (WHO) implemented the MPD-4-QED Programme (henceforth, the “Programme”), a comprehensive electronic data collection system throughout a network of referral-level hospitals in Nigeria. The Programme was designed to systematically gather essential data during the stages of labor, childbirth, and the early postnatal period. The primary goal of this Programme was to streamline the evaluation of the quality of care administered to women and newborns during the perinatal period. The details of the Programme have been previously described.^9^

### Study design, sampling and participants

**Fig. 1:**
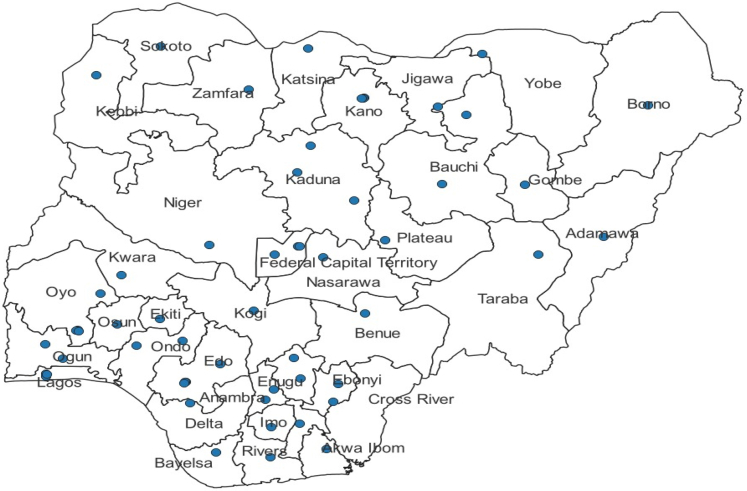
**Map of Nigeria showing participating facilities in the Maternal, Perinatal Database for Quality, Equity and Dignity Programme (MPD-4-QED)**.

The MPD-4-QED network consists of 56 tertiary hospitals that serve as referral centers for neighboring healthcare facilities. Within this network, there are 50 publicly-funded and 6 privately-funded hospitals, distributed across Nigeria's six geopolitical zones: Northcentral, Northeast, Northwest, Southeast, Southsouth, and Southwest. All publicly-funded hospitals providing in-patient services for obstetric and gynaecology services were initially invited to participate (n = 52). Of these, 48 granted consent and participated in the Programme from 2019, an additional two public hospitals began data collection in 2021. While not nationally representative, the facilities were selected to reflect typical referral-level care across Nigeria. Fig. 1 presents a map of Nigeria with the participating facilities.

The study population comprised of all women (and their babies) who were admitted for delivery at or after 28 weeks of gestation between 1 September 2019 and 31 August 2022 in the participating hospitals. Women who were transferred prior to birth were included. Women who were admitted after birth, or at less than 28 weeks gestational age were excluded from this analysis.

### Data collection and electronic capture

An electronic data platform was created by customizing open-source District Health Information Software (DHIS-2), which is the officially endorsed Health Management Information System of the Federal Ministry of Health in Nigeria. This platform gathered data from patient medical records through an electronic case report form, capturing information on the women's sociodemographic information, previous medical history, antenatal records, labor and delivery (including the baby's clinical condition), and immediate postpartum observations.

At each hospital, two Medical Record Officers who underwent training performed daily monitoring of medical records across various hospital units, including the obstetric ward, gynecological emergency unit, birthing/delivery room and operating theatre.

For every woman admitted, data entry commenced upon admission, continued throughout her hospital stay, and was finalized either at the time of discharge or death (whichever was first). The data collection process involved extracting information from patient medical records using a tablet-based case report form that had been specifically designed for this project. To ensure accurate data linkage, a unique identifier was used to connect the information between a woman and her newborn within the database.

The data collected were synchronized in real-time from the internet-enabled tablet device to a secured central cloud-base server. Each participant's data record remained open for a period of 60 days following their admission. This timeframe allowed for adjustments to be made by hospital coordinators to rectify any data entry errors and to complete maternal or perinatal death audit forms when a death had occurred. Several quality assurance processes where in place for the duration of the Programme and are reported elsewhere.^9^

### Outcomes and definitions

#### Sociodemographic information

The sociodemographic characteristics extracted from the medical record were: maternal age, marital status, highest education level attained, woman's occupation and husband/partner's occupation.

#### Clinical information

The clinical information reported for women were: parity, previous caesarean section, previous miscarriage, chronic medical disorder pre-pregnancy, antenatal care and referral status. All characteristics were reported as categorical variables.

#### Indication for cesarean section

Data on indication for caesarean section were collected from medical records and entered into the database. More than one indication could be selected for each woman. The indications were: placental conditions (placenta previa, abruptio placenta), hypertensive disorders (pregnancy-induced hypertension, chronic hypertension, pre-eclampsia/eclampsia), prolonged/obstructed labour (suspected or diagnosed contracted/inadequate pelvis, obstructed labour, failure of labour to progress), fetal distress, failed induction of labour, failed assisted vaginal delivery, cord prolapse, maternal request, macrosomia, intrauterine growth restriction, previous pelvic surgery (including myomectomy), infertility (including IVF baby), elderly primigravida, oligohydramnios/anhydramnios. For indications that could not fit into any of the above categories ‘other’ was selected and considered to have no medical key indication recorded.

#### Robson classification

Women were classified according to the Robson Classification following the instructions in the WHO Robson Implementation Manual^13^ and using the following obstetric variables: parity (nullipara/multipara), previous caesarean section (yes,no), onset of labour (spontaneous, induced, no labour—pre-labour caesarean section), number of fetuses (single/multiple), gestational age (preterm—less than 37 weeks, term—37 weeks or more), fetal lie and presentation (cephalic presentation, breech presentation, transverse lie). For presentation, categories were defined as follows: 1) cephalic (fetal head is the presenting part) included vertex, face or brow, or compound head presentations; 2) breech (fetal buttocks or one foot or two feet are the presenting part) included frank, complete and footling breeches; 3) transverse or oblique lie (fetal long axis is perpendicular or oblique in relation to the mother's long axis) included when fetal shoulder or arm are presenting or there is no presenting part. Women were classified sequentially as described in Supplementary Figure S1.

#### Missing variables

Women with missing data on one or more variables needed for Robson classification were included in an 11th category ‘x–unclassifiable’. To better understand areas for improvement in data collection the number of women with missing information at each stage of Supplementary Figure S1 was examined. The sociodemographic and clinical characteristics of the women in category ‘x-unclassifiable’ were compared to women who were classified into one of the Robson groups to give a better understanding of population differences and to assist interpretation of the findings.

### Analysis

The WHO Robson Implementation Manual was used to lead the interpretation of the use of caesarean section.^13^ We calculated the number and proportion of women in each category of the sociodemographic and clinical variables outlined above. Following the WHO Robson Implementation Manual, we determined: the relative size of each Robson group; the caesarean section rate in each group; the absolute contribution to the overall caesarean section rate (ie, the percentage contributed to the overall caesarean section rate by a particular group); and the relative contribution to the overall caesarean section rate (ie, the absolute contribution expressed as a percentage of the overall rate). The WHO Robson Implementation Manual was used to compare Robson Groups with other reference groups. To understand the variation between facilities the distribution of group size by Robson group across facilities was determined as well as the distribution of caesarean section by Robson group across facilities. In order to gain a more comprehensive insight into the women having caesarean section in each Robson category the indication for caesarean section and induction of labour by Robson group was examined using frequencies and proportions.

### Ethics

The scientific content of the study was approved by the WHO Human Reproduction Programme (HRP) Research Project Review Panel (protocol ID, A65930, 06 May 2018). WHO Ethics Review Committee (ID A65930, 05 June 2018) and the Nigerian National Health Research and Ethics Committee approved the study (ID NHREC/01/01/2007, 05 September 2018). Authorities of all participating hospitals granted written institutional approvals to participate in the programme's data collection, periodic analyses and reporting. Individual level written consent was not required as the study did not involve direct interaction with women or their babies, or interview of medical staff.

### Role of funding source

The funders did not play any role in the study design; in the collection, analysis, and interpretation of data; in the writing of the report; and in the decision to submit the paper for publication. JT,TL, LG had access to the data. The decision to submit the paper for publication was taken by all authors.

## Results

### Sociodemographic and clinical characteristics of study population

Data were analysed for 179,463 women who gave birth at the participating facilities. Most women were 20–35 years (81.4%), were married or co-habiting (98.9%), had secondary or post-secondary education (44.1%, 43.2%), were not gainfully employed (38.4%) and had a husband/partner working in a professional or related category (41.7%). The majority of women were multiparous (53.6%) with 15.4% being grand-multiparous (≥4 previous births), had antenatal care at the same facility as birth (72.2%) and were not referred or self-referred to the participating hospital (86%). For women who had given birth previously 14% had a caesarean section. The sociodemographic and clinical characteristics are presented in Supplementary Table S1.

### Robson classification

The quality of the data was high. When using the WHO Robson Implementation Guide to assess the population we found that compared with the reference obstetric population there were around four-times more multiple pregnancies (Group 8) and three-times more preterm births (Group 10) in our population (Table 1).

**Table 1:** Robson classification in women delivering at 56 referral hospitals in Nigeria (n = 179,463).

Robson classification	Number of CS in group	Number of women in group	Group size (%)^a^	Group CS rate (%)^b^	Absolute group contribution to overall CS rate (%)^c^	Relative contribution of group to overall CS rate (%)^d^
**1: Nulliparous, singleton, cephalic, ≥37 GA, spontaneous**	**4076**	**30,606**	**19.3%**	**13.3%**	**2.6%**	**7.8%**
**2: Nulliparous, singleton, cephalic, ≥37 GA, induced/prelabour CS**	**6702**	**7977**	**5.0%**	**84.0%**	**4.2%**	**12.8%**
2.a: Nulliparous, singleton, cephalic, ≥37 GA, induced	1082	2357	1.5%	45.9%	45.9%	2.1%
2.b: Nulliparous, singleton, cephalic, ≥37 GA, prelabour CS	5620	5620	3.6%	100.0%	100.0%	10.8%
**3: Multiparous (no CS), singleton, cephalic, ≥37 GA, spontaneous**	**3305**	**58,498**	**37.0%**	**5.6%**	**2.1%**	**6.3%**
**4: Multiparous (no CS), singleton, cephalic, ≥37 GA, induced/prelabour CS**	**6263**	**8065**	**5.1%**	**77.7%**	**4.0%**	**12.0%**
4.a: Multiparous (no CS), singleton, cephalic, ≥37 GA, induced	587	2389	1.5%	24.6%	24.6%	1.1%
4.b: Multiparous (no CS), singleton, cephalic, ≥37 GA, prelabour CS	5676	5676	3.6%	100.0%	100.0%	10.9%
**5: Previous CS, singleton, cephalic, ≥37 GA, spontaneous/induced/prelabour CS**	**14,157**	**17,761**	**11.2%**	**79.7%**	**8.9%**	**27.1%**
5.a: Previous CS (one), singleton, cephalic, ≥37 GA, spontaneous/induced/prelabour CS	8338	11,602	7.3%	71.9%	71.9%	16.0%
5.b: Previous CS (more than one), singleton, cephalic, ≥37 GA, spontaneous/induced/prelabour CS	5819	6159	3.9%	94.5%	94.5%	11.1%
**6: Nulliparous with a single breech**	**1606**	**2135**	**1.3%**	**75.2%**	**1.0%**	**3.1%**
**7: Multiparous with a single breech (including previous CS)**	**3024**	**4331**	**2.7%**	**69.8%**	**1.9%**	**5.8%**
**8: Multiple pregnancies (including previous CS)**	**3440**	**6239**	**3.9%**	**55.1%**	**2.2%**	**6.6%**
**9: Single pregnancy, transverse or oblique lie (including previous CS)**	**727**	**759**	**0.5%**	**95.8%**	**0.5%**	**1.4%**
**10: Singleton, cephalic, <37 GA (including previous CS)**	**8921**	**21,875**	**13.8%**	**40.8%**	**5.6%**	**17.1%**
**Total classified in Robson**	52,221	158,246	100.0%	33.0%	33.0%	100.0%
Unclassifiable	6944	21,217	11.8%	32.7%	–	–

a Group size (%) = n of women in the group/total N women who gave birth in the study x 100.

b Group CS rate (%) = n of CS in the group/total N of women in the group x 100.

c Absolute contribution (%) = n of CS in the group/total N of women who gave birth in the study x 100.

d Relative contribution (%) = n of CS in the group/total N of CS in the study x 100.

The overall caesarean section rate was 33.0% in the participating facilities. The largest relative contribution to overall caesarean section rate were women with term pregnancy and previous caesarean section (Group 5, 27.1%), followed by women with preterm pregnancy (Group 10, 17.1%). Women without previous caesarean section who had induced labour or those who had a pre-labour caesarean section also made substantial contributions to overall caesarean section rate (Group 2–12.8%; Group 4–12.0%).

Through applying the WHO Robson Classification implementation guide we could better understand caesarean section rates by Robson group. Box 1 provides a summary of the interpretation for each Robson category. The guide suggests that 20–35% of women in Group 2 (nulliparous, singleton, cephalic, term) will require caesarean to improve maternal and fetal outcomes. In the current study 84.0% of women had a caesarean section in Group 2. The main indications for pre-labour caesarean section (Group 2b) were hypertensive disorders (18.9%) and suspected inadequate/contracted pelvis (13.2%) (Supplementary Table S2). One-third of women in Group 2 had induction of labour and the indications were postdated pregnancy (Group 2a—1109/2204, 50.3%) and hypertensive disorders (373/2204, 16.9%). In women who had induction of labour (Group 2a) around half had an intrapartum caesarean section (1082/2357, 46%). The main indications for caesarean section after induction were failed induction of labour (57.9%) and prolonged or obstructed labour (40.4%). The most common method for induction was misoprostol followed by intracervical (Foley) catheter and oxytocin infusion (Supplementary Table S4).

Box 1 **Interpretation of type of population, CS rates, indication for CS and indication for labor induction using WHO Robson Implementation Manual for the women admitted to the participating hospitals**

^∗∗^MCS reference population was the population of the MCS with relatively low CS rates and, at the same time, with good outcomes of labour and childbirth.

A high proportion of multiparous women (singleton, cephalic, term) without previous CS (Group 4) had a caesarean section (77.7%) compared to the 15% of women suggested in the WHO Robson Implementation Manual. Among women having pre-labour caesarean section in Group 4b the main indications were hypertensive disorders (15.0%), placental conditions (11.9%) and fetal distress (8.1%) (Supplementary Table S2). In women who had induction of labor (Group 4a) one-quarter had an intrapartum caesarean section (587/2389, 24.7%) with main indications failed induction of labour (56.7%) and prolonged labour (28.5%).

### Variation across facilities

**Fig. 2:**
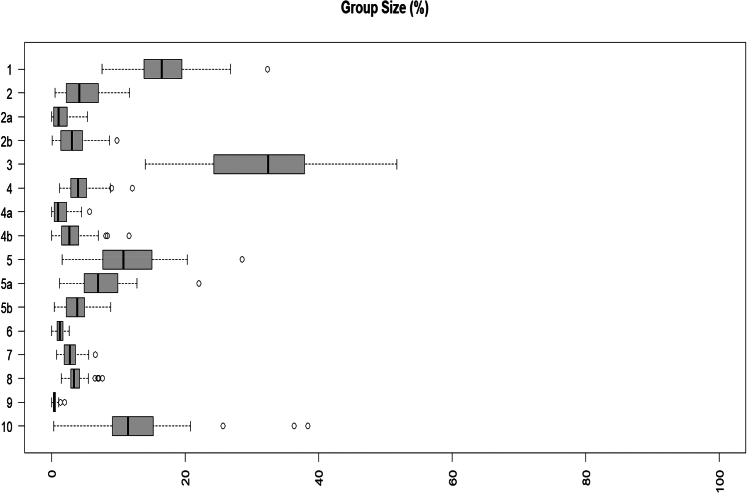
**Distribution of group size by Robson group across facilities**.

**Fig. 3:**
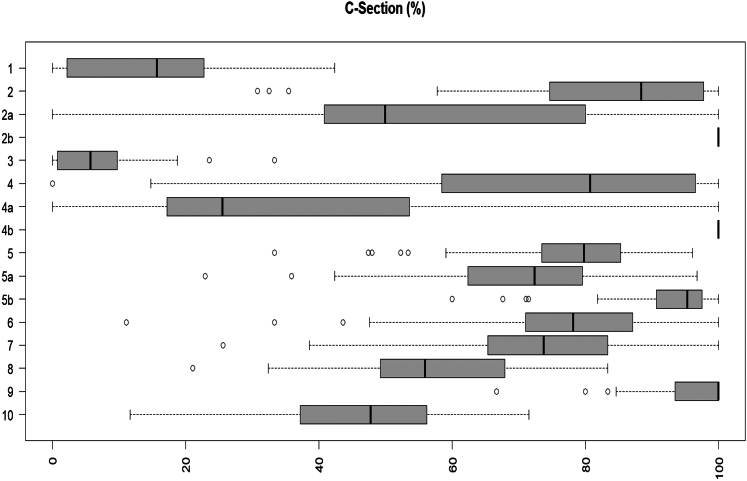
**Distribution of caesarean section by Robson group across facilities**.

Fig. 2 shows the variation in Group size (%) across facilities by Robson group. Group 3 (multiparous women with term pregnancy and without previous caesarean section) with spontaneous labour) had the greatest variation (IQR 23%–38%). The size of Groups 6, 7, 8, 9 had the least variation between facilities. Fig. 3 shows boxplots for caesarean section rate by Robson Group in the participating facilities. All groups present large variability between hospitals. Group 2a and 4a (nulliparous and multiparous women with term cephalic pregnancy who had labour induced, respectively) had the greatest variation in the proportion of caesarean section across facilities (Groups 2a (IQR 40–80%), Group 4a (IQR17-53%)). This indicates large variation in caesarean section after induction. Supplementary Figure S2 shows between-hospital variation for pre-labour caesarean section rate, induction of labour with final mode of delivery vaginal birth, and induction of labour with final mode of delivery caesarean section.

### Missing data

There were 21,217 (11.8%) women with missing data on at least one variable needed for the Robson classification. Of the women who could not be classified 13,263 (64.8%) were due to missing gestational age, a further 4450 (23.3%) were due to missing presentation of the baby and a further 1936 (10.1%) were due to missing information on previous caesarean section. In addition 1568 had missing data on mode of delivery. Women who had missing information were younger, had lower education, were not gainfully employed and did not have antenatal care. As detailed in our companion paper,^9^ missingness was not random and reflects routine data limitations.

## Discussion

Using the WHO Robson Implementation Guide, we found the data to be of high quality. Compared to the reference population in the WHO Manual, our population had about four times more multiple pregnancies and three times more preterm births. Around one-third of women across the participating facilities in Nigeria had a caesarean section. A large proportion of women with single, term, cephalic pregnancy (without previous caesarean section) had a pre-labour caesarean section which was coupled with a low induction of labour rate in the same obstetric population.

The overall rate of caesarean section was similar to global estimates from the WHO Multi-country Survey which included 66 health facilities in 22 countries. In Nigeria, previous facility estimates vary from 11.5% to 17.6%.^11^^,^^14^ The population caesarean section rate in Nigeria is estimated at 2.7% which is in stark contrast to the 30% facility rate observed in this study.^1^ While there is no consensus on the ideal population-level caesarean section rate due to the complexities of population differences and ability to ascertain individual need for caesarean section, global data suggests a cesarean section rate of 10–15% is reasonable to avoid unnecessary caesarean sections and associated risks, but ensure caesarean is performed when medically necessary.^15^, ^16^, ^17^ The juxtaposition between the low population caesarean section rate and high facility rate observed in the current study may indicate challenges with access to this life-saving procedure contributing to high mortality in Nigeria.

The current study is the first to apply the Robson Classification in multiple facilities in Nigeria which can assist to understand how caesarean over-use and under-use can coexist. One striking finding was the relatively large proportion of women with single term cephalic pregnancy (without previous caesarean section) who had a pre-labour caesarean section, far more than other settings with published results from Robson. The WHO multi-country survey (WHO MCS) found less than half of women in these groups had caesarean section (23.7%–39.9%).^18^ Interestingly, a qualitative synthesis of evidence on women's birth preferences found less than two percent of Nigerian women expressed a preference for caesarean section. The study reported a reluctance of women to request or accept caesarean section. Mothers from low-socioeconomic backgrounds or those who are primary caregivers were particularly concerned about the economic implications of undergoing a cesarean section.^19^ On the contrary a qualitative synthesis across 28 countries found that health workers often reinforce women's preferences for caesarean section, particularly in the absence of clinical indications, citing the convenience of scheduling, predictability in the birth timeline, and systemic constraints. More exploration is needed into the high caesarean section rate which may be in conflict with women's preferences.

Coupled with the high pre-labour caesarean section rate was a low induction of labour rate. In the WHO MCS 4.5–15.5% of women had an induction of labour, while a cross-sectional study in Latin America of 120 hospitals reported a prevalence of 11.4%.^18^ In the current study around half of women had an caesarean section after labour induction compared to one-third of women in the WHO MCS. The high pre-labour caesarean section rate coupled with the low successful induction rate may indicate providers preferences for pre-labour caesarean over induction of labour perhaps driven by a lack of national, harmonized clear clinical guidelines, inadequate staff resources preventing monitoring of women and/or, concerns on the quality of inducing agents, among others.^20^ The main indications for pre-labour caesarean were hypertensive disorders and suspected contracted/inadequate pelvis. Training and support to offer women with hypertensive disorders (including pre-eclampsia) safe induction of labour could be explored. While inadequate pelvis and cephalopelvic disproportion are contraindications for induction of labour improved assessment of women with suspected contracted/inadequate pelvis through clinical pelvimetry or radiography may enable better selection of women who require pre-labour caesarean section. A survey of 252 pregnant women in Nigeria found that concerns prior to induced labour were fear of caesarean section birth (16.6%), labour pains (15%) and adverse fetal or maternal outcome (4.8%). After induction 71.4% of women were satisfied with the process.^21^ Many women with hypertension are referred from outside facilities and have already spent several days on admission prior to labour.^22^ The long referral pathway (often including significant challenges with transport) as well as concerns about the stress and time taken for induction coupled with the risk of failed induction may contribute to women's and providers preferences for pre-labour caesarean section. The large number of women been referred from peripheral facilities may also contribute to the high overall caesarean section rate.

We also observed inter-facility variation. Facilities that attempted fewer inductions also had a higher intrapartum caesarean section rates. This suggests that these facilities may not be well-supported in appropriate selection of women for induction of labour and/or do not have adequate support during inductions which require continuous monitoring of labour progress and fetal monitoring. To increase successful induction women require a supportive environment with emotional support, encouragement and a positive and calming environment. Approaches such as continuous labour companionship and midwife-led care have been associated with higher proportions of vaginal births, higher maternal satisfaction and lower health-care costs relative to control groups in high-income countries.^23^^,^^24^ It is important to explore in more detail the drivers of unsuccessful induction and the potential avenues to increase successful labour induction such as by better selection of women who could benefit from induction in Nigeria and lower-resource settings.

The study has some limitations. Notably, the population was drawn from referral-level facilities and does not reflect the general population. In addition, some women could not be classified into any Robson group due to missing data. Despite these limitations, he Robson classification system can be applied using simple information routinely collected. The Robson classification is used to better understand caesarean section use, however it can also reveal information about the population of women accessing the facilities. It was an interesting observation that overall the women in our study had high fertility, were multiparous, had more multiple pregnancies and more preterm births compared to other settings. This is the first time data from a country with such a population profile has had Robson classification applied. Countries with high fertility such as Niger, Mali, Chad and Angola may expect to find similar findings as the current study.

Lastly, optimizing the use of caesarean section requires more than clinical interventions alone. WHO has recently produced guidelines on antenatal and intrapartum care and these include recommendations on some of the clinical interventions that reduce caesarean section and improve other outcomes for mother and baby. Non-clinical interventions are underpinned by behavioural and psychosocial drivers encompassing financing and care-provision models, system integration, and environmental and resourcing conditions. Interventions at the system level will likely require changes in organizational culture, insurance reforms, facility staffing models, specific goals for caesarean section and induction rates, and targeted financial strategies. Fundamental to this is investment in the training of health professionals. A multi-faceted and locally tailored approach is required addressing women's and health professional concerns, as well as health system and financial factors.

## Contributors

The implementation of this project was a collaborative effort of a large number of academic staff, hospital personnel and researchers from 56 referral-level Nigeria hospitals − The Maternal and Perinatal Database for Quality, Equity and Dignity (MPD-4-QED) Network. TL, JT, APB conceived the study. TL drafted the protocol for the analysis of the database with substantial input from JT, APB. TL and LG prepared the statistical analysis plan and led all statistical analysis. TL, JT and LG all accessed and verified the underlying data. All authors reviewed and interpreted the data at a workshop convened by WHO. TL led and coordinated the writing of the manuscript. The writing committee (JT, ANO, AA, EE, DCN, LG, AA, SMD, AA, RI, OEJ, IAU, AO, EA, OS, AA, AIN, LM, PA, HG, CC, JI, AO) provided feedback on the first draft. TL and APB revised and consolidated the final manuscript. All authors and named members of MPD-4-QED Network had an opportunity to revise the manuscript for intellectual content and approved it for publication. The manuscript represents the views of the named authors only and does not reflect the views of MSD for Mothers, the UNDP/UNFPA/UNICEF/WHO/World Bank Special Programme of Research, Development and Research Training in Human Reproduction Programme (HRP) or the World Health Organization.

## Data sharing statement

All relevant data are within the manuscript and its Supporting Information files.

## Editor note

The Lancet Group takes a neutral position with respect to territorial claims in published maps and institutional affiliations.

## Declaration of interests

The authors declare no competing interests.
