# Supplementary material for: Analyzing caesarean sections through the Robson classification in Nigeria: a prospective nationwide study in referral level facilities
Source: eClinicalMedicine. 2025 Sep 1;87:103427. doi: 10.1016/j.eclinm.2025.103427 (PMC12424238; doi:10.1016/j.eclinm.2025.103427)
Supplement: Supplementary Tables and Figures [file mmc1.docx]

# Supplementary Figures

# **Figure S1. Flow chart for the classification of women in the Robson Classification (according to WHO Robson Implementation Manual)**


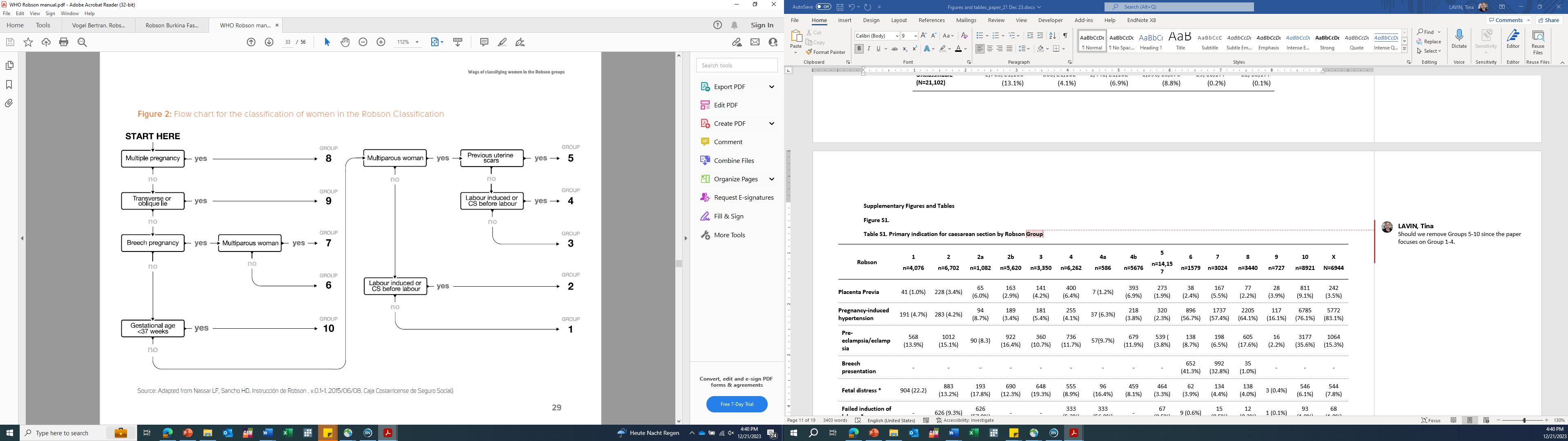


# **Figure S2. Mode of birth by on-set of labour (spontaneous, labour induction) across facilities in women with single, cephalic, term, pregnancy.**

A – Robson Groups 1 & 2.

Spontaneous vaginal: spontaneous on-set of labourwith vaginal mode of birth; Spontaneous CS: spontaneous on-set of labour with caesarean section mode of birth; Pre-labour CS: pre-labourcaesarean section mode of birth; Failed induction: induced on-set of labour with caesarean section mode of birth; Successful induction: spontaneous on-set of labourwith vaginal mode of birth.

B – Robson Groups 3 & 4

**.**

Spontaneous vaginal: spontaneous on-set of labour with vaginal mode of birth; Spontaneous CS: spontaneous on-set of labour with caesarean section mode of birth; Pre-labour CS: pre-labour caesarean section mode of birth; Failed induction: induced on-set of labour with caesarean section mode of birth; Successful induction: spontaneous on-set of labour with vaginal mode of birth.

# Supplementary Tables

# **Table S1. Sociodemographic characteristics of the population in 56 referral hospitals in Nigeria (N=****179,463)**

| **Characteristic** | **n/N (%)** |
| --- | --- |
| **Maternal Age** |  |
| ≥9 and <20 years | 6,413/179,459 (3.6%) |
| 20-35 years | 146,098/179,459 (81.4%) |
| >35 years and ≤55 | 26,948/179,459 (15.0%) |
| Unknown | 4 |
| **Marital status** |  |
| Single | 1,786/178,936 (1.0%) |
| Married/Cohabiting | 176,943/178,936 (98.9%) |
| Separated/Divorced | 179/178,936 (0.1%) |
| Widowed | 28/178,936 (0.0%) |
| Unknown | 527 |
| **Woman's education level** |  |
| No formal education | 15,342/165,478 (9.3%) |
| Primary | 5,688/165,478 (3.4%) |
| Secondary | 72,984/165,478 (44.1%) |
| Post-secondary or more | 71,464/165,478 (43.2%) |
| Unknown | 13,985 |
| **Woman's occupation** |  |
| Not gainfully employed | 67,246/174,959 (38.4%) |
| Professional/technical/managerial/Civil servant/administrative/clerical | 38,322/174,959 (21.9%) |
| Sales/trading | 41,704/174,959 (23.8%) |
| Farming/agricultural work/skilled manual/unskilled manual | 11,986/174,959 (6.9%) |
| Other or Unknown | 15,701/174,959 (9.0%) |
| Unknown | 4,504 |
| **Husband's occupation** |  |
| Not gainfully employed | 1,735/174,064 (1.0%) |
| Professional/technical/managerial/Civil servant/administrative/clerical | 72,663/174,064 (41.7%) |
| Sales/trading | 48,150/174,064 (27.7%) |
| Farming/agricultural work/skilled manual/unskilled manual | 16,347/174,064 (9.4%) |
| Other or Unknown | 33,176/174,064 (19.1%) |
| Single | 1,993/174,064 (1.1%) |
| Unknown | 5,399 |
| **Parity** |  |
| Nullipara (0) | 55,670/179,441 (31.0%) |
| Multipara (1-3) | 96,210/179,441 (53.6%) |
| Grand multipara (4 or more) | 27,561/179,441 (15.4%) |
| Unknown | 22 |
| **Previous caesarean section** |  |
| No | 94,594/174,724 (54.1%) |
| Yes | 24,460/174,724 (14.0%) |
| Nulipara | 55,670/174,724 (31.9%) |
| Unknown | 4,739 |
| **Previous miscarriage** | 46,066/175,349 (26.3%) |
| Unknown | 4,114 |
| **Chronic medical disorder (pre-pregnancy)** | 12,855/177,496 (7.2%) |
| Unknown | 1,967 |
| **Antenatal Care** |  |
| No antenatal care or informal setting | 20,016/177,482 (11.3%) |
| ANC at the same health facility | 128,115/177,482 (72.2%) |
| ANC at another health facility | 29,351/177,482 (16.5%) |
| Unknown | 1,981 |
| **Referral status** |  |
| Not referred or self-referred | 154,260/179,457 (86%) |
| Referred from public or private hospital | 24,008/179,457 (13%) |
| Referred from informal setting | 1,189/179,457 (0.7%) |
| Unknown | 6 |
| n/N (%) | |

# **Table S2. Primary indication for caesarean section by Robson Group ^**

| **Robson** | **1**  **n=4,076** | **2**  **n=6,702** | **2a**  **n=1,082** | **2b**  **n=5,620** | **3**  **n=3,305** | **4**  **n=6,263** | **4a**  **n=587** | **4b**  **n=5,676** | **X**  **N=6944** |
| --- | --- | --- | --- | --- | --- | --- | --- | --- | --- |
| **Placental conditions** | 119 (2.9%) | - | 19 (1.8%) | 305 (5.4%) | 328 (9.2%) | 698 (11%) | 24 (4.1%) | 674 (11.9%) | 484 (7.0%) |
| **Hypertensive disorders +PE** | 723 (17.7%) | 1241 (18.5%) | 176 (16.3%) | 1065 (18.9%) | 503 (15.2%) | 943 (15.1%) | 89 (15.2%) | 854 (15.0%) | 1207 (17.4%) |
| **Prolonged/obstructed labour/suspected inadequate pelvis^&^** | 2311 (56.7%) | 1181(17.6%) | 437 (40.4%) | 744 (13.2%) | 1281 (38.8%) | 435 (6.9%) | 167 (28.5%) | 268(4.7%) | 3289 (47.4%) |
| **Fetal distress** | 904 (22.2%) | 883 (13.2%) | 193 (17.8%) | 690 (12.3%) | 648 (19.6%) | 555 (8.9%) | 96 (16.4%) | 459 (8.1%) | 544 (7.8%) |
| **Failed induction of labour** | - | 626 (9.3%) | 626 (57.9%) | - | - | 333 (5.3%) | 333 (56.7%) | - | 68 (1.0%) |
| **Failed assisted vaginal delivery** | 12 (0.3%) | 3 (0.0%) | 3 (0.3%) | - | 12 (0.4%) | 2 (0.0%) | 2 (0.3%) | - | 42 (0.6%) |
| **Cord prolapse** | 19 (0.5%) |  | 3 (0.3%) | 15 (0.3%) | 58 (1.8%) | 78 (1.2%) | 5 (0.9%) | 73 (1.3%) |  |
| **Maternal request** | 62 (1.5) | 401 (6.0%) | 11 (1.0%) | 390 (6.9%) | 46 (1.4%) | 336 (5.4%) | 7 (1.2%) | 329 (5.8%) | 128 (1.9%) |
| **Macrosomia** | - | 345 (5.2%) | - | 345 (6.1%) | - | 364 (5.8%) | - | 364 (6.4%) | 59 (0.9%) |
| **IUGR** | - | 58 (0.9%) | - | 58 (1.0%) | - | 23 (0.4%) | - | 23 (0.4%) | 12 (0.2%) |
| **Previous pelvic surgery** | - | 199 (3.0%) | - | 199 (3.5%) | - | 81 (1.3%) | - | 81 (1.4%) | 27 (0.4%) |
| **Infertility** | - | 187 (2.8%) | - | 187 (3.3%) | - | 48 (0.8%) | - | 48 (0.9%) | 19 (0.3%) |
| **Primigravida>35 years of age** | - | 228 (3.4%) | - | 228 (4.1%) | - | - | - | - | 16 (0.2%) |
| **Oligohydramnios/Anhydramnios** | 62 (1.5%) | 157 (2.3%) | 11 (1.0%) | 146 (2.6%) | 84 (2.5%) | 108 (1.7%) | 2 (0.3%) | 106 (1.9%) | 101 (1.5%) |
| **Other** | 461 (11.3%) | 1528 (22.8%) | 35 (3.2%) | 1493 (26.5%) | 686 (20.8%) | 2008 (32.1%) | 29 (4.9%) | 1895 (33.3%) | 1222 (17.6%) |

^ more than one indication per woman can be present; ^&^ includes suspected c**ontracted/inadequate pelvis** identified prior to labour

#

# **Table S3. Indications for induction***

| **Robson Classification** | **Postdated pregnancy** | **Hypertensive disorders** | **Diabetes during pregnancy** | **Intrauterine growth restriction (IUGR)** | **Intrauterine fetal death** | **Maternal request** | **Pre-labour rupture of fetal membranes** | **Congenital anomaly of the fetus** | **Other** |
| --- | --- | --- | --- | --- | --- | --- | --- | --- | --- |
| **2a (N=2357)** | 1,109/2,204 (50.3%) | 373/2,204 (16.9%) | 18/1,968 (0.9%) | 34/1,968 (1.7%) | 70/1,968 (3.6%) | 18/1,968 (0.9%) | 131/1,968 (6.7%) | 4/1,968 (0.2%) | 300/2,204 (13.6%) |
| **4a(N=2389)** | 1,166/2,309 (50.5%) | 376/2,309 (16.3%) | 27/2,138 (1.3%) | 30/2,138 (1.4%) | 112/2,138 (5.2%) | 29/2,138 (1.4%) | 103/2,138 (4.8%) | 8/2,138 (0.4%) | 375/2,309 (16.2%) |

*Proportion may add to more than 100% because more than one indication can be recorded.

# **Table S4. Methods for induction**

| **Robson Classification** | **Misoprostol** | **Intracervical (Foley's) catheter** | **Membrane sweeping** | **Oxytocin** | **Other prostaglandins** | **ARM** | **Other** |
| --- | --- | --- | --- | --- | --- | --- | --- |
| **2a (N=2,357)** | 1,512/2,068 (73.1%) | 443/2,068 (21.4%) | 36/2,068 (1.7%) | 275/2,068 (13.3%) | 1/2,068 (0.0%) | 14/2,068 (0.7%) | 76/2,068 (3.7%) |
| **4a(N=2,389)** | 1,499/2,220 (67.5%) | 566/2,220 (25.5%) | 30/2,220 (1.4%) | 312/2,220 (14.1%) | 0/2,220 (0.0%) | 11/2,220 (0.5%) | 70/2,220 (3.2%) |
